# Lithium Ions as Modulators of Complex Biological Processes: The Conundrum of Multiple Targets, Responsiveness and Non-Responsiveness, and the Potential to Prevent or Correct Dysregulation of Systems during Aging and in Disease

**DOI:** 10.3390/biom14080905

**Published:** 2024-07-25

**Authors:** David A. Hart

**Affiliations:** Department of Surgery, Faculty of Kinesiology, McCaig Institute for Bone & Joint Health, University of Calgary, Calgary, AB T2N 4N1, Canada; hartd@ucalgary.ca

**Keywords:** lithium, biomolecular regulation, evolution, molecular co-factors, preclinical models, neuroregulation, immune regulation, human heterogeneity

## Abstract

Lithium is one of the lightest elements on Earth and it has been in the environment since the formation of the galaxy. While a common element, it has not been found to be an essential element in biological processes, ranging from single cell organisms to *Homo sapiens*. Instead, at an early stage of evolution, organisms committed to a range of elements such as sodium, potassium, calcium, magnesium, zinc, and iron to serve essential functions. Such ions serve critical functions in ion channels, as co-factors in enzymes, as a cofactor in oxygen transport, in DNA replication, as a storage molecule in bone and liver, and in a variety of other roles in biological processes. While seemingly excluded from a major essential role in such processes, lithium ions appear to be able to modulate a variety of biological processes and “correct” deviation from normal activity, as a deficiency of lithium can have biological consequences. Lithium salts are found in low levels in many foods and water supplies, but the effectiveness of Li salts to affect biological systems came to recent prominence with the work of Cade, who reported that administrating Li salts calmed guinea pigs and was subsequently effective at relatively high doses to “normalize” a subset of patients with bipolar disorders. Because of its ability to modulate many biological pathways and processes (e.g., cyclic AMP, GSK-3beta, inositol metabolism, NaK ATPases, neuro processes and centers, immune-related events, respectively) both in vitro and in vivo and during development and adult life, Li salts have become both a useful tool to better understand the molecular regulation of such processes and to also provide insights into altered biological processes in vivo during aging and in disease states. While the range of targets for lithium action supports its possible role as a modulator of biological dysregulation, it presents a conundrum for researchers attempting to elucidate its specific primary target in different tissues in vivo. This review will discuss aspects of the state of knowledge regarding some of the systems that can be influenced, focusing on those involving neural and autoimmunity as examples, some of the mechanisms involved, examples of how Li salts can be used to study model systems, as well as suggesting areas where the use of Li salts could lead to additional insights into both disease mechanisms and natural processes at the molecular and cell levels. In addition, caveats regarding lithium doses used, the strengths and weaknesses of rodent models, the background genetics of the strain of mice or rats employed, and the sex of the animals or the cells used, are discussed. Low-dose lithium may have excellent potential, alone or in combination with other interventions to prevent or alleviate aging-associated conditions and disease progression.

## 1. Purpose of the Review

Lithium is one of the lightest elements and is found in high concentrations throughout the universe. On Earth, lithium is found in many chemical forms, including carbonates, in brines, and in other types of deposits. While lithium has been used in the treatment of bipolar disorders since the early reports of Cade [1] and follow-up studies in Europe (discussed in [2]), it has more recently gained prominence as a central component of lithium-ion batteries, used in electric automobiles. This has led to a resurgence in lithium mining throughout the world, as well as the introduction of lithium into the environment via battery recycling and processing.

While prominently represented in the environment, lithium ions are not essential co-factors in biological systems (discussed in [3,4,5]), although deficiencies in lithium can lead to compromised health (discussed in [6]). In contrast, sodium, potassium, calcium and magnesium are essential co-factors in multiple biological systems, and others such as iron, zinc, and copper play a role in some specific systems. Thus, lithium ions appear to have been isolated from being incorporated as an essential element during the evolution of increasingly complex biological systems. 

While not essential in biological systems, lithium ions appear to serve a biological regulator role in some systems, and thus, it can play a somewhat unique role in biology as a modulator of dysfunction and “chaos”. This review will discuss how lithium ions function as a regulator of diverse biological systems, the systems involved, and then, focusing on two examples, the possible mechanisms involved in such a regulation. With the advent of electric vehicles and the need for lithium as a component of batteries, there has been an increase in lithium mining and the recycling of such batteries, both with the potential to increase lithium levels in water supplies and food (discussed in [7]). Thus, the role of lithium in the regulation of biological systems is becoming a very relevant issue and a better understanding of its role as a modulator of molecular processes is critical to increasing awareness.

## 2. Introduction

Elemental lithium is very reactive with water and thus needs to be handled with care. In contrast, lithium ions readily form carbonates and are distributed in brines around the world, and low to modest amounts of lithium salts can be found in most water supplies. Lithium exists as two isotopes, ^7^Li and ^6^Li, with ^7^Li as the most prominent isotope at ~95% and ^6^Li a minor isotope at ~5%. ^7^Li can be isolated from ^6^Li and assessed for biological activity separately ([8]; discussed in [9]). As ^7^Li has an ionic radius similar to that of magnesium and ^6^Li has an ionic radius similar to sodium, and both magnesium and sodium are involved in differing biological processes [i.e., sodium ions in the Na/K membrane transporter and magnesium as a cofactor in several enzymes], one might expect that the two isotopes of lithium may have differing biological effects.

While Li ions are abundant in the environment, including sea water, where early life forms likely evolved, this element is not an essential element for the complex lifeforms existing today [5]. This includes humans, wherein Li is considered an important micronutrient, but not an essential element. Thus, while very abundant, it was apparently excluded from playing an essential role in biological processes (discussed in [3,4,5]). Alternatively, because of its abundance and unique chemical properties, it may serve a unique role in the regulation of biological processes, as deficiencies can result in compromised health (discussed in [6]).

The ”modern” era of using Li salts likely can be attributed to its use in the 1940s and early 1950s, one with very positive outcomes and the other with negative outcomes. Regarding the former, based on the work of Australian John Cade, who reported that administration of Li salts calmed guinea pigs and subsequently showed that such salts were effective in treating patients with bipolar disorder (discussed in [1,10,11]). However, some authors say that Cade “rediscovered” Li salt effects and they were discovered 50 years earlier (discussed in [11]). Irrespective of who discovered Li salt’s effects on patients with bipolar disorder, their use in these conditions became the standard for decades and continues today. In the treatment of bipolar disorder patients with lithium salts, it has been noted that not all patients are responsive to therapeutic doses of this intervention [12,13,14]. In fact, only 30% of patients can be classified as responders. Thus, there appears to be genetic variables that influence the responsiveness to what are borderline toxic concentrations. Attempts to identify the genes involved have not yet led to definitive associations.

In contrast, the use of Li salts with negative outcomes has also influenced Li salt use in the USA, as well as supported the understanding of the role of Li in biological systems. In response to emerging evidence that too much NaCl was detrimental for cardiovascular health, the substitution of LiCl for NaCl in the diet was proposed and implemented as a “non-toxic” alternative [15,16]. It soon became apparent that such an approach was toxic, and it led to multiple deaths. This led the FDA in the USA to declare LiCl a poison, and the practice was discontinued in disgrace. This negative outcome indicates that excessive amounts of Li salts had a negative impact on biological systems and thus, it was biologically active, but the mechanisms were still not defined in detail [15]. The second result from this failed “experiment” was that there was a reluctance to use Li salts in the treatment of bipolar disorder in the USA, although it was widely studied and used in Europe.

While the outcome of Li salt use as a substitute for NaCl was a clear failure, its successful use in the treatment of bipolar disorder in patients was a stimulus from several perspectives for a more detailed investigation of Li salt’s effects on other biological systems, with the emergence of a better understanding of its broad significance in biology and its role(s) as a modulator or regulator of biological systems. It plays a very critical role in controlling variation in system integrity due to genetics, epigenetics, or biological heterogeneity in humans, as well as in other vertebrates and invertebrates and plants. Thus, in this context, it can be used to better understand how it could work as a regulator, as well as serving as a tool to perturb systems, providing insights into biological regulation.

The effect of Li salts on cells in vitro exhibits a property termed “hormesis” [17], where low doses can lead to positive effects, but higher doses to negative effects. This property of hormesis by lithium may also be exerted in vivo, where high doses (hundreds of mg/day) are required to treat bipolar disorder, but very low doses (<1–10 mg/day) may be sufficient to function as a microregulator of biological processes and/or prevent dysregulation. This dichotomy in activity does not follow the usual pharmacological paradigm for most drugs, and likely would lead to complicated clinical trials. However, since Li salts cannot be patented per se, there is often little incentive for the pharmaceutical industry to support such trials, but perhaps private foundations might find such support more appealing.

## 3. Molecular Mechanisms of Action of Li Ions

While lithium is not an “essential” element in any reported enzyme or protein activity, it has been reported to exhibit a biomodulatory effect on a spectrum of molecules and biological processes. This spectrum is quite complex, with a number of primary and secondary effects that appear to be too complicated to decipher [3,4,6,18,19,20,21,22,23,24]. The molecular targets of lithium include the following: Na/K ATPases, ion channels, GSK-3beta, inositol metabolism components, cytoskeleton components, neurotransmitters, Wnt/beta-catenin, miRNAs, enzymes associated with DNA methylation and demethylation, and other epigenetic modifications, such as histone deacetylase activity (discussed in [25,26,27]), adenyl cyclase, cyclic AMP, to name several (Table 1; also see Table 3 of reference [6] for several additional details). In many of the in vivo studies, the investigations have led to the belief that one of the primary targets of lithium is GSK-3beta, which then leads to other secondary effects on other molecules. An example of this relates to the effects of lithium on the cytoskeleton, where the inhibition of GSK-3beta leads to alterations in the cytoskeleton [28] versus a potentially primary effect [29]. 

Another set of targets of lithium action are reported to be ion channels [26,27]. These ion channels may be calcium-, sodium-, or potassium-specific [26,27,30,31]. Several of these are reported to exhibit hyperexcitability in the neurons of bipolar disorder patients [26,27,30,31]. Such excitability appears to be different in lithium responsive patients from either normal individuals or lithium non-responsive bipolar disorder patients [30,31].

With a multitude of potential targets available for lithium, the interpretation of in vivo results is often complicated, particularly when different doses of lithium salts are investigated, and different routes of administration are employed in the studies. While this breadth of potential lithium targets is supportive of the concept that lithium serves a role as a general modulator of biological dysfunction [32], it presents a conundrum for researchers in that different primary targets are relevant in different tissues and different types of dysregulations.

However, the number of potential targets for lithium action is also likely dependent on the concentrations of lithium salts used for studies, and the primary role is associated with those targets associated with low levels [3,6]. While this concept would pertain to the environmentally available concentrations of lithium, or possibly those associated with certain natural springs with elevated lithium levels from dissolved salts, the fact that very high, nearly toxic levels of lithium are required to exert an effect in subsets of patients with bipolar disorders implies that other variables must be at play in such psychiatric disorders. Given the commonality of many of the targets for lithium action, it is unclear why and how resistance even to high doses can occur in many bipolar disorder patients, where only 30% or less are responders [12,13,14]. This difference in responder vs non-responder phenotype may be associated with genetics [13], but how such genetic influences are translated to specific molecular mechanisms remains to be elucidated. Alternatively, lithium-responsive and lithium-resistant bipolar disorders may be different sub-types of the conditions [31]. Furthermore, how resistance even to high doses of lithium salts in some bipolar disorder patients is manifested without affecting the systems impacted by low environmental doses remains to be elucidated. Interestingly, an increased risk of developing biological side effects has been noted in some bipolar disorder patients treated with high doses of lithium (Table 2), but many patients tolerate the high doses. As high doses would never have been encountered via environmental levels during evolution, this heterogeneity also requires further understanding.

**Table 2:** Tissue targets for increased risk for side effects of lithium treatment.

System Affected	Reference
Thyroid	Hassaguerre and Vantyghem, 2022 [33]
	Phelps and Coskey, 2024 [34]
Parathyroid	Misfsud et al., 2020 [35]
	Hassaguerre and Vantyghem, 2022 [33]
Kidney	
Diabetes insipidus	Ohlund et al., 2018 [36]
Renal toxicity	Rej et al., 2016 [37]
	Davis et al., 2018 [38]
Muscle/tremors	Ohlund et al., 2018 [36]
	Gelman et al., 2024 [39]
Heart	Frassati et al., 2004 [40]
	Singh et al., 2016 [41]
	Mehta and Vannozzi, 2017 [42]
Brain	Young, 2009 [43]
Bone	Tannirandorn and Epstein, 2000 [44]
	Wagner et al., 2011 [45]
	Hamstra et al., 2024 [46]

The indicated references are all reviews on the subject and are representative rather than all-inclusive. Additional systems may be affected following exposure to toxic levels of lithium or increased effects on the systems listed; for example, after bariatric surgery.

Relevant to the issue of the relationship of lithium salts, dosage, and molecular target issues, there have been a number of reports that indicate lithium appears to be able to influence conditions other than bipolar disorders, often at levels reported to be much lower than those used in bipolar disorders (Table 3). Several of these conditions relate to diseases or injuries involving the brain (Table 3). Therefore, the discussion below will focus on those involving neural conditions, as well as aspects of autoimmunity as examples. However, the details regarding which molecular targets of lithium are the primary targets for many of these conditions remain to be elucidated, as the chronic administration of the lithium may lead to primary and secondary effects. Furthermore, rodents such as mice and rats tolerate higher doses of lithium salts than humans do, so this may also impact the translation of findings in such experimental models to humans. 

**Table 3:** Dose-dependent effects of lithium on human-relevant diseases.

Condition/Disease	Lithium Levels	Species	Reference
Bipolar Disorders	300–500 mg/day	Human	Hamstra et al., 2023 [46]
Alzheimer’s Disease			
Water Supplies	1–3 mg/day	Human	Muronaga et al., 2022 [47]
Oral Supplement	300 ug/day	Human	Nunes et al., 2013 [48]
Nanolithium	40 ug/kg/day	Human	Rat Guilliot et al., 2024 [49]
Parkinson’s Disease	15–150 mg/day	Human	Guttuso et al., 2023 [50]
	Variable	Several	Singulani et al., 2024 [51]
Huntington’s Disease	150–300 mg/day	Human	Danivas et al., 2013 [52]
	150 mg/day	Human	Serafini et al., 2016 [53]
	0.5–5.0 mM	Mice	Scheuing et al., 2014 [54]
	1.5–3.0 mEq/kg	Rats	Scheuing et al., 2014 [54]
Prion Disease	160 ug/kg	Mice	Relano-Gines et al., 2018 [55]
	16 mg/kg	Mice	Relano-Gines et al., 2018 [55]
Brain Trauma	1.0–5.0 mM	Rodents	Richard, 2024 [56]
Hematology	0.3–1.0 mM	Human	Focosi et al., 2009 [57]
	1.0–5.0 mM	Rodents	Focosi et al., 2009 [57]
Autoimmunity	2.0–8.0 mg/day	Mice	Hart, 2016 [9]

Selected articles representing the breath of the potential disease impact of lithium and the variations in effective doses. Several of the articles cited are recent reviews, to which the reader is referred for details of the specific area.

## 4. Lithium Salts and Non-Bipolar Disorders: Patients and Preclinical Mammalian Models

### 4.1. Human Neural Conditions and Disorders

While lithium is well known for its track record in treating bipolar disorders, it has more recently been investigated for efficacy in other human conditions (Table 3; reviewed in [51,54,58,59,60,61,62,63,64,65,66,67]). The potential conditions in which lithium may have efficacy include Alzheimer’s, Parkinson’s, and Huntington’s diseases, traumatic brain injury and stroke. For most of these conditions there are very few clinical trials, so this is an emerging area of research. However, one trial of note is the study by Nunes et al. [48], who reported that microdose lithium treatment was effective in stabilizing cognitive impairment in patients with Alzheimer’s disease. Exposure to lithium in patients with mild cognitive impairment can have long term benefit, even well after a clinical trial has ended [68]. This area has also been reviewed recently by Guillot et al. [49]. Such studies are also supported by epidemiological investigations of incidence of Alzheimer’s disease and drinking water lithium levels. Muronaga et al. [47] reported that higher levels of lithium in drinking water in Japan led to a lower incidence of Alzheimer’s disease, but mainly in females and not males. This research focused on individuals >65 years of age, so the females were post-menopausal, and this population makes up ~70% of cases (discussed in [69]). The role of lithium in epilepsy has been investigated, but the beneficial effects of the treatment are not yet clear (reviewed in [70]).

While details regarding how lithium may be influencing such conditions still remain to be elucidated, several reports have postulated that it works via GSK-3beta-related systems to exert a neuroprotective effect [20,71], possibly via the regulation of apoptosis and autophagy [72,73]. From a considerable body of literature of studies with cells, the treatment of cells with lithium salts leads to the downregulation of pro-apoptosis molecules and the upregulation of anti-apoptosis molecules (discussed in Section 5), so it may be exerting some of its neuroprotective effects via the regulation of these molecules, possibly in concert with brain-derived neurotrophic factor (BDNF). In addition, lithium ions can also influence neurotransmitter systems [74,75], so may also be able to foster other aspects of neuronal function. Relevant to this point, in the review by Dean and Scarr [75], it is discussed that exposure to mood stabilizers such as lithium leads to alterations in the release of neurotransmitters such as glutamate, dopamine and serotonin, and affects pre-synaptic pathways.

### 4.2. Preclinical Mammalian Models of Lithium Action

To further understand the molecular mechanisms involved in lithium actions and the breadth of its influence on biomolecular processes, a number of animal models have been developed, ranging from invertebrates to mammalian models, particularly rodent models. Many of these provided several insights into lithium actions at a variety of doses and in a variety of conditions. In the discussion below, some examples of mammalian models have been chosen to illustrate their potential to elucidate features of lithium responsiveness and resistance.

#### 4.2.1. Models of Neural Diseases/Disorders

The effectiveness of lithium exposure in the treatment of bipolar disorders, and the associations of lithium levels with dementia incidence, has led to the development of a variety of preclinical models of neural degeneration syndromes to assess whether lithium might be effective and how it may work (reviewed in [21,23,76]). Such models range from fruit flies, zebra fish and rodents, with most models shown to have effective targets for lithium action (discussed in [23]). Even in normal C57BL/6 male mice, low-dose lithium appears to influence GSK-3beta in a region-specific manner [77].

As neural degeneration conditions such as Alzheimer’s disease may take years or even decades to develop and progress to overt dementia, many researchers have turned to rodent models (reviewed in [78,79,80]). As detailed in Cuello et al. [79], as of 2018 there have been 160 rodent models studied (156 mouse and 4 rat). These authors discussed the fact that many of the models were focused on the rarer forms of familial neurodegeneration rather than sporadic forms of such diseases. 

Two of the more widely used mouse models are the 3XTg-AD mouse [81,82] and the McGill-Thy1-APP model [80]. Both of these are transgenic models on a C57BL/6 background, fully or partially. In the 3XTg-AD model, exposing mice to LiCl in the chow diet led to a number of therapeutic benefits [83], correcting several of the alterations induced by the transgenes leading to the Alzheimer’s disease-like phenotype. It has been suggested this effect of lithium action occurs via the inhibition of GSK-3beta, and this possibility has been supported by the studies of Croft et al. [84], who reported that a different inhibitor of GSK-3beta was also able to modify tau abnormalities in brain slices from 3XTg-AD mice. In a third model of Alzheimer’s disease, the TgCRND8 model, leptin has been reported to be effective in ameliorating pathology and cognition (reviewed in [85]), and leptin and lithium appear to share some molecular targets, such as GSK-3beta and PI-3 kinase [86], and interrelationships. Low-dose lithium treatment in a double transgenic mouse model of Alzheimer’s disease on a C57BL/6 genetic background has also been shown to have efficacy regarding pathology and cognitive ability [87].

While the number of rat models of Alzheimer’s disease is much lower than mouse models, the use of the McGill-R-Thy1-APP and TgF344-AD rat models does offer some advantages over those models in mice. Such comparisons have been extensively reviewed by Cuello et al. [79]. These models are transgenic models expressing relevant human genes in the brain of the rats (reviewed in [79,88]). Both rat models are albino, but the Wistar is outbred while the TgF344-AD rat is inbred on the F344 background. Information regarding the original coat color of these two rats could not be found.

In the McGill-R-Thy1-APP rat model, exposure to very low concentrations of lithium (~400 times lower than that used for the treatment of bipolar disorder patients) leads to beneficial effects (decreased pathology and improved cognition) (reviewed in [79]). Such microdose lithium formulations are effective in both male and female animals in this model of Alzheimer’s disease [89]. Such findings are consistent with the studies of Nunes et al. [48], who reported that microdoses of lithium stabilized cognitive impairment in patients with Alzheimer’s disease.

Lithium effectiveness has apparently not been investigated to the same extent in the other rat model of Alzheimer’s disease, namely the TgF344-AD rat. However, a histone deacetylase inhibitor RG2833 has been shown to exhibit efficacy in this model [90], and lithium is reported to be a histone acetylase inhibitor, so perhaps it may have efficacy in this model. 

There are also a number of other rodent models in which lithium has been shown to have efficacy or has been investigated. These include rat models of Parkinson’s disease [51,62,63,91], traumatic brain injury [59], stroke [66], Huntington’s disease [54], and prion disease [55]. In many of these models, lithium has been shown to exert a neuroprotective effect, possibly due to its role as an anti-apoptotic agent (discussed below in Section 5).

#### 4.2.2. Autoimmune Mouse Models of Systemic Lupus Erythematosus (SLE)

For many years, it was reported that patients receiving lithium salts for bipolar disorder experienced alterations to components of the immune system (reviewed in [3,4,18,92,93]). The cells affected included lymphocytes and granulocytes/polymorphonuclear leukocytes (PMN). Lithium treatment led to the release of PMN from the bone marrow and alterations to hematopoiesis (i.e., granulocytosis and lymphopenia), as well as enhancing the activity of monocytes (reviewed in [43]). Based on such findings, a series of studies were undertaken with several murine/mouse models of SLE. These are mainly genetic models and do not require any induction, appearing in a defined timeframe, and several exhibit sex differences in the severity of the disease. 

In the BXSB model of murine SLE, disease occurs mainly in male mice [94,95], in contrast to human SLE which predominantly develops in females [96,97]. In this model, the mice develop a range of autoantibodies to nuclear components, splenomegaly, and lymphadenopathy, and the male animals die fairly early in life from nephritis, with ~90% deaths by 8 months of age (reviewed in [98]). The treatment of male BXSB mice with daily intraperitoneal injections of doses of LiCl, that were non-toxic in multiple strains of mice [8,99], led to the accelerated deaths of the animals [100]. Thus, in this model, exposure to modest levels of LiCl appeared to accelerate the nephritis, developing as a consequence of the autoimmune process. Further attempts to define the molecular basis for this acceleration were not undertaken, refraining from characterizing any shifts in autoantibody profiles or any details regarding where and how the lithium exposure might be affecting the kidney. Such studies may be of interest, as males make up a minority of SLE patients, but they often suffer more severe complications.

A second widely used SLE murine model is the MRL-lpr mouse (reviewed in [98]). In this model, both males and females develop an autoimmune disease with renal involvement. However, this model not only develops severe splenomegaly and a spectrum of autoantibodies, but also a renal disease with some features unlike those arising in human SLE [98]. This model also exhibits some involvement of neuropsychiatric SLE [101], but not as much as the induced models [102]. The treatment of female MRL-lpr mice with LiCl led to a modest improvement in the survival of the females, survival which was dependent on the age when the LiCl was initiated (reviewed in [9,103]). Starting the LiCl injections at an early age was most effective at enhancing survival, indicating that once the disease process was firmly established, exposure to LiCl became less and less effective. This finding supports the conclusion that disease induction is dependent on specific molecular mechanisms sensitive to lithium suppression, but once the SLE-like disease become established or chronic, a different set of molecular mechanisms, lithium independent, become operative. The findings also imply that the initiation of the SLE-like disease can vary somewhat, and not all animals develop features of the condition at the same age. As survival was the primary outcome of the study of lithium effects in this model, it is not known if the treatment affected any neuropsychiatric manifestations of the disease process.

A third commonly used genetic model of murine SLE is the NZB/W F1 model (reviewed in [98]). This SLE model is an F1 between the NZB strain (black coat color) and the NZW strain (white coat color) and shares many characteristics with human SLE. In this model, female F1 mice develop a fulminant disease much earlier than the slower onset, progress and milder disease in male F1 mice, and ~90% die of renal failure in the first year [98]. In addition to developing a spectrum of autoantibodies (i.e., anti-DNA and anti-chromatin) and renal disease, these animals also develop neuropsychiatric manifestations [102,104], cardiovascular alterations [105], and multiple organ involvement [106,107]. A recent report [108] detailed similarities and differences between this model and human SLE. 

The treatment of 8–10 week-old female NZB/W mice with 4 mg LiCl/day for >1 year led to the long term survival of ~50% of the mice (reviewed in [9,109,110]). Starting the LiCl treatment before puberty (6–8 weeks of age in mice) did not improve the percentage of long-term survivors, but delaying the initiation of treatment to >12 weeks of age led to fewer long-term survivors (reviewed in [9]). However, administering 4 mg LiCl twice a day (morning and evening) to 8–10 week-old females led to the long-term survival of 80% of the mice >18 months of age (reviewed in [9,111]). Interestingly, the cessation of treatment at 18 months of age led to a re-activation of the lupus nephritis and the death of these long-term surviving animals. Thus, the lithium treatment did not stop the disease, but prevented the consequence of the disease process on the kidney. Interestingly, the treatment of NZB/W F1 mice with theophylline, an inhibitor of adenyl cyclase, has also been reported to prolong survival and decrease renal damage in this model [112], findings consistent with the report of Rao et al. [113], indicating a potential mechanism of lithium action on GSK-3beta and adenyl cyclase.

Further studies with the NZB/W model also revealed additional information: (1) using LiCl in the drinking water rather than injecting it led to the induction of diabetes insipidus/polydipsia but did not prolong survival of females [114]; (2) injecting purified ^6^LiCl or ^7^LiCl enhanced the survival of females, but some subtle differences were noted [115,116]; (3) the treatment of male NZB/W mice with 4 mg LiCl/day did not enhance survival [9]; (4) the treatment of female NZB/W mice did not alter their autoantibody profiles [117] but did alter renal gene expression profiles [118]. 

During the aging of untreated NZB/W female mice, it was noted that the animals put on significant weight before they succumbed to their SLE-like disease. This added weight nearly doubled their initial weight at 8–10 weeks of age and was almost entirely subcutaneous fat (reviewed in [9,109,110]). The treatment of female mice with 4 mg LiCl/day completely blocked the development of these subcutaneous fat deposits. The relationship of this effect of lithium salt treatment on the development of obesity and the observed effects on the survival and maintenance of renal function is not known. As obesity can lead to metabolic syndrome which can impact renal function [119], and it exhibits racial differences [120], it is possible that the protection of renal function in the lithium-treated female mice could be due to direct effects on the kidney, or indirect effects via an influence on obesity development, which then impacts the kidney, or a combination of both. In C57BL/6 mice (which have a black coat), this may also be mediated by the cytokine irisin [121]. However, this explanation of a potential relationship between obesity and renal effects may not explain everything about the ability of lithium to affect survival, as 100% of treated females did not develop obesity, but the long-term survival in some studies was only 50%. Thus, additional studies are required to better understand how lithium treatment in this SLE model exerts a protective effect. Furthermore, none of the reported studies investigated the effects of lithium salt treatment on the cardiovascular system or the brain, both areas that are reported to be altered in this model [102,105].

Lingering questions from the NZB/W studies relate to the various mechanisms for the outcomes that were assessed, how to integrate the findings in a coherent manner mechanistically, and how treatment with lithium salts was involved in modifying disease progression in this model. Regarding the onset and progression of fat deposits and the development of obesity in these mice, it has been noted that the NZB/W mice have elevated blood levels of leptin, and that leptin administration to such mice accelerated autoantibody production and renal disease [122]. Similarly, SLE patients also have elevated leptin levels (discussed in [122]). However, leptin is reported to influence energy metabolism to inhibit the development of obesity in rodent and animal models [123,124,125,126,127]. As the kidney has receptors for leptin [128], it may be related to hypertension developing in obesity [129], ability to interact with the sympathetic nervous system [130], and is metabolized in the kidney [131]. Leptin may be functioning via the melanocortin pathway or via GSK-3beta (a lithium sensitive pathway), either peripherally or centrally via the hypothalamus. However, one should not lose sight of the fact that several aspects of the physiologic roles of leptin differ between mice and humans [132], findings that may influence the translation of findings from mouse models to humans. In spite of such limitations, the finding of leptin-related events in lithium responsiveness in patients can still be very relevant [133,134,135,136].

#### 4.2.3. Induction of Diabetes Insipidus/Polydipsia in Different Strains of Mice: Genetic Influences

The treatment of patients with lithium salts often leads to the induction of diabetes insipidus/polydipsia, or excessive urination [36,137,138,139]. This effect is due to the impact of lithium directly or indirectly on the collecting ducts of the kidney [140,141,142,143]. In a subset of patients, lithium treatment leads to a vasopressin-resistant state [137], and relevant to this discussion, it has been reported that GSK-3beta mediates the renal response to vasopressin by modulating cyclooxygenase-2 [144] and adenyl cyclase [113]. As both GSK3beta and adenyl cyclase have been implicated in lithium effects, this may be the mechanistic link (discussed in [113]). Further, some reports indicate there are sex differences in the induction of polydipsia (females > males) [145] and the other side effects of such treatments [146]. As only a subset of patients developed polydipsia while on lithium treatment, this implied that there was heterogeneity in the population regarding this response, and thus, the same thing might occur in mice and potentially have influenced the response patterns in the autoimmune models discussed earlier. Conversely, the prolonged use of lithium in patient populations can lead to an increased risk of loss of kidney function and nephropathy in a subset of patients [147,148]. Thus, the kidney appears to be a site for lithium modulation, but the responses are heterogeneous in the treatment population. Based on the involvement of the kidney in lithium responsiveness, a number of animal models have been investigated regarding the diabetes insipidus/polydipsia [149] and other features of lithium exposure [150] to elucidate the molecular mechanisms of action.

To address the potential issue of heterogeneity in lithium responsiveness between inbred mouse strains, female mice from a number of strains were obtained from Jackson Laboratories (NZB, NZW, A/J, C57B/6, DBA, BALB/c, C3H/HeJ, albino C57Bl/6, C57BL/6 X BALB/c F1) and exposed to graded doses of intraperitoneal injections of LiCl ([99,151] reviewed in [9]). In response to 4 mg LiCl/day, the induction of overt polydipsia between strains was NZB = C57BL/6 >>> DBA = C3H/HeJ >> BALB/c. Interestingly, C57BL/6 X BALB/c F1 mice exhibited a response pattern similar to that of the NZB/W F1 mice, indicating that the presence of the BALB/c contribution diminished the responsiveness of the C57BL/6 contribution. This dose of LiCl was toxic for all of the A/J mice and they all died of unknown causes. Of note is that both the NZB and C57BL/6 strains have a black coat, while the BALB/c and A/J mice have a white coat and the C3H/HeJ and DBA mice have an intermediate coat color. Furthermore, the NZB and C57BL/6 mice responded well to lower doses of LiCl (2 mg LiCl), while the other strains assessed did not. A comparison of the induction of polydipsia in the C57BL/6 and albino C57BL/6 mice indicated no significant differences in response to lithium, indicating that melanin synthesis was not involved in the response phenotype. As MRL-lpr mice have a white coat and BXSB mice have a brown/agouti coat, and the kidney appears to be a target in LiCl-mediated survival of autoimmune mouse models, these results may offer at least a partial explanation why the lithium treatment of NZB/W was effective. That is, the NZB/W mouse is an F1 between a very lithium responsive strain (NZB) and a resistant strain (NZW), and apparently, the NZB strain contribution may be contributing a lithium-responsive phenotype. However, the F1 mice were modestly resistant to the induction of polydipsia with 4 mg LiCl/day, but resistance could be overcome somewhat with 2× 4 mg LiCl/day (discussed in [9,152]). Thus, when treatment of the F1 mice was initiated at 8–10 weeks of age with 4 mg LiCl/day, only 50% were long term survivors (reviewed in [9]), possibly implying that there was a genetic variable influencing long-term survival. Increasing the dose of LiCl to 2 x 4 mg/day led to the long-term survival of 80% of the NZB/W mice, so higher doses may overcome the influence of the more resistant parental strain (NZW). Unfortunately, these studies were only performed with female mice, so any sex differences in this regard remain to be determined.

The notion that a genetic influence was playing a role in the long-term survival of F1 mice treated with 4 mg LiCl/day was further investigated with F2 combinations [152]. These studies indicated that the female mice generated from such crosses exhibited quite a variable induction of polydipsia (11–39 mL water/day), and that those derived from some litters were quite responsive and other litters were more resistant. Thus, there may be complex genetic contributions and interactions leading to the induction of polydipsia and long-term survival following treatment with LiCl in these mice. In addition, in rats, the route of administration of the lithium salt makes a difference in the induction of diabetes insipidus/polydipsia (IP > SQ = via a gastric tube) [153], so this is a variable that must be considered when assessing responsiveness and resistance.

There are two important points that one can make from the above information. The first is that there are parallels between the mouse findings and reports in the literature regarding human responses to lithium. There are a number of reports indicating that there are differences between Caucasians and Black people at the level of kidney function [154], neuropharmacology [155,156,157,158], lithium response patterns [159] and lithium metabolism, suggesting that Black people may require lower doses than Caucasians [160]. While incomplete at several levels, the data may indicate some parallels between black vs white mice and humans of differing ethnicities. 

A second point raised by these studies in mice is that there are strengths and weaknesses in working with inbred mice, and these mouse studies emphasize the weakness of working with only one strain of mice and then attempting to translate the findings to humans. As humans are very heterogeneous (discussed in [161]), and the mice assessed are inbred, the findings with a single mouse strain may only be relevant to a small subset of humans and thus appear to not translate very well.

## 5. Effect of Li Salts on Cells In Vitro

As mentioned above, Li salts on cells in vitro exhibit hormesis [17]. That is, the exposure of cells to low doses of Li salts such as chloride or carbonate can lead to positive effects on a variety of biological parameters, while higher doses inhibit the same or similar processes. Thus, Li salts are effective in the treatment of a subset of patients with bipolar disorder, but exhibit a very different profile of effectiveness at levels more aligned with the endogenous levels supplied from water sources, plant food, or animal food. However, it should be noted that endogenous levels can vary greatly depending on geographic location, as Li salts in water supplies and levels in food sources can vary (discussed in [17]). 

The body of literature regarding the use of cells in vitro is quite broad, and for many of the studies the use of lithium salts is mainly as a tool to better understand the biomolecular pathways involved in responsiveness to an intervention. Furthermore, many studies used cell lines that have been in culture for decades while others were freshly isolated from preclinical animals or were white blood cells from humans. In addition, some reports indicate the cells are cultured in the presence of xenogenic serum (i.e., porcine cells in fetal bovine serum) or in a different medium. All of these variables can influence outcomes and make it difficult to compare in vitro and in vivo findings. Finally, this also includes the sex of the cells. As cells can reflect the sex of the source of the cells [162,163,164], it is important to know and report the sex of the cells used in a study, as lithium may impact males and females differently in vivo (i.e., as discussed in earlier sections). Many reports still do not indicate the sex of the cells, in spite of initiatives to require such disclosure [162]. This dilemma also influences the interpretation of outcomes of adding sex hormones to a cell when the sex of the cell is not reported. An example of this is the report by Sandhu et al. [165], in which Madin–Darby canine kidney cells (MDCK cells; male source) were used and the effect of estrogen and testosterone on cell function was assessed, but the sex of the cells was not mentioned.

While there are many limitations in the study of lithium effects on cells in vitro, depending in part on whether they are immortalized cell lines or fresh cells, as well as the culture conditions and the interpretation of the findings, some specific examples of potentially relevant studies will be presented in the below discussion. 

### 5.1. Cells of the Brain and Neural Cells

A large body of literature using cells derived from the brain has been developed over the past several decades (reviewed in [21,166,167]). In vitro, lithium has been reported to enhance the neuronal differentiation of neural progenitor cells [168], which can then be used for transplantation in preclinical models. Lithium treatment led to the upregulated expression of brain-derived neurotrophic factor. It did not significantly influence proliferation but did significantly impact differentiation of the cells. Following the treatment of astrocytes from C57BL/6 mice, Rivera and Butt [169] reported that lysyl oxidase and peroxisome proliferator-activated receptor-gamma were targets of lithium action. Lithium treatment in vitro and in vivo also led to elevated levels of bcl-2, an anti-apoptotic molecule, which may exert a neuroprotective effect via this mechanism [170,171]. Using embryonic neurons from Wistar rats, DePaula and Forlenza [24] reported that hippocampal neurons were more sensitive to lithium than cortical neurons were. In a study of rat neurons in vitro, Dwivedi and Zhang [22] reported that lithium treatment led to an increased expression of bcl-2 and bcl-XL and the downregulation of the pro-apoptotic molecules’ bas, bad and caspase-3, as well as the epigenetic modification of the BDNF gene promoter. Interestingly, bcl-2 was also upregulated following the treatment of the porcine renal proximal tubule cell LLC-PK1 [172], so this may be a common effect of lithium to promote cell survival in both the brain and the kidney. Relevant to this point is also the report that lithium can protect neuronally differentiated PC12 cells, a rat cell line [173], from apoptosis-inducing stimuli [170]. This protective effect of lithium against neurodegenerative diseases may be related to this molecular action. It has been proposed that this effect of lithium could be due to its ability to modify the activity of GSK-3beta and the Wnt signaling pathway [174], but the details remain to be elucidated. The possibility that mood stabilizers such as lithium could work via antiapoptotic mechanisms has been raised by Chuang [175] previously.

Lithium has also been reported to exert a neuroprotective effect via the modulation of the microRNA miR-1906 with lithium-enhanced EV, derived by bone marrow MSC and assessed on astrocytes, glial cells and neurons derived from male C57BL/6 mice in vitro, and in an in vivo stroke model in C57BL/6 mice [176]. Whether this effect is also observed in other inbred strains of mice, particularly those with a different coat color, remains to be determined. Finally, further to a potential link between lithium action and leptin levels discussed above, leptin has also been reported to inhibit GSK-3beta downstream of an effect on the kinase AMPK in the female human neuroblastoma cell line SH-SY5Y [177]. As leptin is also pleiotropic, with several diverse effects on biological systems, there may be some crosstalk with lithium effects via GSK-3beta [133].

### 5.2. Cells of the Immune System

The treatment of patients with lithium has been known to lead to alterations in the immune system and the cells of the immune system (reviewed in [3,4,18,178,179,180]). In patients with bipolar disorder, lithium treatment can result in an increase in polymorphonuclear leukocytes (PMN) and their degranulation products [180]. In cancer patients treated with chemotherapy resulting in depressed hematopoiesis, lithium treatment-enhanced PMN counts increase, but not those for lymphocytes [179]. In vitro exposure to lithium salts can also result in enzyme release from PMN [181], which is isotope (^6^Li or ^7^Li) independent [182]. In vitro, lithium can influence lymphocyte gene expression and is reported to influence that of bipolar patients in a unique manner [183]. The exposure of lymphocytes from preclinical models that are stimulated with mitogens, bacterial lipopolysaccharide, and antigens can lead to enhanced stimulation (reviewed in [3,4,18,184]). The details of the molecular mechanisms underpinning such effects of lithium salts have not been clearly defined, but what is evident is that such exposures can influence the properties of lymphocytes in vitro and PMN in vivo and in vitro.

### 5.3. Kidney Cells

A subset of patients treated with lithium salts for psychiatric conditions develop diabetes insipidus/polydipsia [137,139,144], and some can develop nephrotoxicity with prolonged use [185,186,187]. Given the role of renal tubule cells in some of these lithium effects on the kidney, studies with renal tubule cells or kidney cells in vitro from a variety of species have been performed (discussed in [172]). 

Using two male porcine kidney cell lines, the PK (15) renal tubule cell line and the proximal tubule LLC-PK1 cell line, Lucas et al. [172,188] reported that studies of the lithium effects with these two cell lines led to very different outcomes. When LiCl, at doses of 5–20 mM, was assessed for effects on the cells, the exposure of PK (15) cells led to induction of apoptosis-related genes and apoptosis of the cells, while similar doses of lithium with the LLC-PK1 cells were well tolerated, and the induction of anti-apoptosis-related molecules occurred. Thus, from the PK (15) studies, one may conclude that this may be a mechanism leading to nephrotoxicity, with long-term high-dose lithium use in lithium-responsive bipolar disorder. In contrast, the results with the LLC-PK1 cells may be related to those lithium-responsive bipolar patients receiving high doses of lithium but who do not develop nephrotoxicity. However, such studies may also reflect the heterogeneity of cell responses when cells are immortalized in long-term in vitro culture. Such alternatives could be further explored using a number of fresh human renal cells from biopsies from both patients responsive and non-responsive to lithium.

### 5.4. Summary and Unresolved Issues Regarding Molecular Effects Lithium Responsiveness and Resistance In Vivo

Based on the above discussion, much of the mechanistic understanding regarding the molecular targets for lithium action has come from the study of cells in vitro, where culture conditions can be somewhat controlled. While such studies are very informative, cells in vitro have been removed from their in vivo regulatory interactions implemented via autocrine, paracrine and endocrine interactions, as well as neuro-control mechanisms. In addition, the concentrations of lithium that can be used in vitro to elicit mechanistic insights have often been in excess of levels that can be employed in vivo.

Based on the human and animal studies, it is not yet clear how and why exposure to lithium in vivo, even at near toxic levels in bipolar disorder patients, causes only a subset to respond well to the treatment. Based on ion channel excitability studies of cells from lithium responders and lithium non-responders, it was suggested that the response pattern may reflect different disorders [31]. 

While there is increased risk for side effects in bipolar disorder patients, some are more common than others (Table 2); therefore, there is heterogeneity in the occurrence of such side effects for reasons that remain to be elucidated. Some of this heterogeneity is likely related to genetic variation based on the diabetes insipidus/polydipsia studies in the series of inbred mouse strains discussed above. As humans are very heterogeneous at the genetic level, it is also likely that genetics play a critical, but currently unknown role in lithium responsiveness in vivo.

Finally, given the plethora of reported molecular targets for lithium action (Table 1), it still remains to be elucidated how lithium is potentially effective in treating disease states (Table 3), but is less effective in modifying non-dysregulated biological systems. Granted, a subset of bipolar disorder patients treated with lithium experience side effects, and numerous inbred strains of mice, particularly those with a black coat color, can develop diabetes insipidus/polydipsia when treated with lithium salts, although they do not have any overt kidney disease and the drug does not “cause” biological dysregulation. This conundrum also needs to be resolved by future research. 

Of note, the ability of lithium to affect dysregulated biological processes but not the analogous normally regulated process has been encountered in a different research environment previously. In the study of abnormal skin wound healing in a pig model, the treatment of red Duroc pigs who exhibit abnormal skin wound healing, with a mast cell stabilizer that inhibits degranulation of mast cells, led to a normalization of wound healing [189]. However, in the similar treatment of Yorkshire pigs that heal skin wounds normally, the drug had no detectable effect, even though mast wells were detected in the wound sites [189]. Therefore, analogous to lithium, in this scenario, the drug was only effective in the dysregulated state.

How and why such discriminating abilities are manifested by lithium should be the topic of future research, as a better understanding of this phenomenon may have general application.

## 6. Lithium and Pregnancy: Risks for Adverse Events to the Mother and Fetus

For nearly all of the above discussion of the influence of lithium salts on biological processes, particularly the in vivo and clinical studies, the recipients have been adolescents or adults. Given the spectrum of molecular targets for the lithium actions discussed throughout this review, in particular the potential role as a modulator of epigenetic modifications to DNA, the topic of the potential effects of lithium during pregnancy is also an area for investigation. 

For females with bipolar disorder who are being treated successfully with lithium, stopping treatment during pregnancy could increase the risk of a relapse of the condition, and thus maintaining lithium treatment during pregnancy and lactation may be necessary for the health of the mother. However, lithium has been reported to affect 236 genes in responsive patients (discussed in [190]) and induce epigenetic alterations in DNA from blood cells [191,192,193,194,195,196]. While some studies reported general methylation alterations, the report by Zafrilla-Lopez [196] indicated that specific genes were modified, and these included genes such as GSK-3beta, a molecule known to be influenced by lithium. While these studies were focused on blood cell DNA changes, it still remains to be determined whether there are other tissue-specific epigenetic alterations that occur in lithium-responsive individuals. Relevant to this point, in the human neuroblastoma cell line SK-N-SH, lithium treatment led to the epigenetic modification of a large number of sites, many more than valproate or carbamazepine [197]. In the rat model, the lithium treatment of rats for 30 days led to epigenetic effects on the leptin receptor gene in the hippocampus [198].

While there are multiple targets for change in the pregnant female who had been treated with lithium, there are a few adverse effects reported for such females [199,200,201,202,203]. These include miscarriages and early delivery. Changes in responsiveness to lithium during pregnancy has not been reported. However, such studies have not distinguished an increased risk for such alterations between those inherent to the psychiatric condition versus the treatment with lithium [199].

Lithium can be delivered from the mother to the fetus, as it can cross the placental barrier [204] and can also be delivered to the neonate via breast milk [205], as well as entering into the developing brain of preclinical models, such as rats [206]. Thus, during these critical stages of development [207], an early maturation due to exposure to medications during pregnancy could have detrimental consequences [208,209]. 

The exposure of infants to lithium via breast milk has not been reported to affect general growth and development. However, some studies have reported an increased risk for neurodevelopmental alterations, but most of these were in preclinical models (reviewed in [210]). In humans, most studies reported normal neurodevelopment after in utero exposure to lithium, but some reported delays in neuromotor development. An increased risk for cardiac malformations has also been reported following in utero exposure to lithium via maternal use [211]. In mice, in utero exposure to lithium has been reported to disrupt embryo development [212], and in C57BL/6 mice, lead to cardiac malformations that can be reversed by folate [213]. It has also been reported that knocking out the lithium target GSK-3beta in C57Bl/6 mice leads to cardiac malformation and is lethal, while knocking out GSK-3alpha does not lead to a cardiac phenotype [214], possibly indicating the target of high-dose maternal lithium levels leads to an increased risk for cardiac malformation in humans via this target. In the study by Rogers and Varnuza [212], the concentrations of LiCl that induced the disruption of embryos was quite high and beyond the usual therapeutic dose. Furthermore, it was reported that disruptions were greater in the LTXBO strain (with C57BL/6 involvement in the genetic background) than in the CD-1 strain (an outbred mouse with a white coat).

Many of the studies focused on investigating the effect of lithium exposure on the fetus have attempted to address overt histologically detectable influences, fetal death or specific target organ abnormalities, and some planned studies in humans will also focus on early events [215]. However, it is possible that the influence of lithium exposure during fetal development may be more subtle and only lead to affects later in life, particularly if the primary mechanism of influence is via epigenetic mechanisms. Relevant to this point are the reports of in utero exposure to lithium leading to a risk of autism spectrum disorder development (discussed in [216,217,218]). Also relevant are the findings of altered neuroendocrine–cytokine axis components in neonatal rats after maternal exposure to lithium during pregnancy [219].

Given the number of potentially relevant targets via which lithium salts could impact fetal development, it is curious that the risks posed are quite modest in humans (discussed in [220]), and in the case of autism spectrum disorder, the risk is also modest, possibly indicating there is only a subpopulation of people who harbor a risk that is elaborated by exposure to lithium. This may imply that if the major influence of lithium is indeed on the development of centers in the brain and via epigenetic mechanisms, and if so, the influence may not be overtly detected until much later in life. Alternatively, during evolution, currently unknown mechanisms to protect the fetus from such outside regulatory mechanisms as lithium were developed, and thus the fetus is “protected” from the influence of lithium in this unique environment. This “protective” mechanism may also be evident in that only ~30% of patients with bipolar disorders are responsive to lithium therapeutics. However, it should be kept in mind that this 30% is responsive to high doses of lithium that are inhibitory, and at low doses, it can be stimulatory (the “hormesis” effect; [17]). However, if such a protective mechanism is a reality, it is difficult to imagine how such a mechanism would arise via evolution, since one would likely never encounter high lithium levels in nature and they have only been attained for therapeutic purposes for the past <100 years!

Therefore, while a mechanism may be in place, and arising through serendipity to prevent or inhibit negative effects of lithium on fetal development and successful generation of offspring, low levels of lithium in the environment could still have a positive effect on fetal development and serve as a biomolecular modulator of any potentially negative events arising via stochastic mechanisms. Interestingly, during embryonic development, the organ levels of lithium derived from the environment are maximal during the first trimester, and then decline to levels that are only ~33% of the first trimester levels by the third trimester (reviewed in [6]). 

This concept of lithium needing to be available during fetal development could be assessed using lithium deficient preclinical models to determine the potential of lithium deficiency to exert negative effects on reproductive processes, fetal development and neonatal survival and maturation. Some of the studies in this area have been reviewed by Schrauzer [6]. Some negative effects of lithium deficiencies on both pregnancy and the successful development of the fetus and survival of the neonate have been described, but some are species-specific (i.e., rat or goat) and there is little evidence of such deficiencies in humans. Interestingly, some of the studies reviewed by Schrauzer [6] indicated that even in the lithium-deficient state, some organs in the fetus were able to maintain their lithium levels (i.e., adrenals, pituitary, and hippocampus), potentially indicating that the fetus has mechanisms to regulate lithium accumulation, which serves specific biomolecular purposes. Thus, high levels of maternal lithium do not induce extensive alterations to the fetus, but low levels appear to serve unique purposes during fetal development. While the mechanisms related to how and why the fetus accumulated low levels of lithium into specific organs during gestation are not known in any detail, this could be a productive area for future research activity.

## 7. Conclusions and the Way Forward

From the studies and concepts discussed, it is apparent that lithium ions can exert biomodulatory effects on a variety of biological systems. These may be organ-specific or cell-specific, and a variety of species can be influenced—not always in a similar manner, but in ways that complement the evolutionary diversity of humans and other species. As such, lithium ions can serve as both a tool to study biological regulation in normal circumstances and under conditions where disease/pathology is evident. Depending on the dose assessed and the species, lithium ions can contribute to the induction of the dysregulation of normal homeostatic mechanisms or correct or influence the outcomes of systems already dysregulated due to other factors, such as in affective disorders or autoimmune diseases in murine models.

The question then arises as to whether supplementing the diet with low doses of lithium ions could be used to metabolically “tweak” biological systems before they become overtly dysregulated, such as during the aging process when there is loss of regulation that could lead to disease, involving molecular mechanisms that could be influenced by lithium ions (discussed in [46]). Given the plethora of potential molecular sites of action for lithium ions (i.e., adenyl cyclase, NaK ATPase, inositol kinases, histone deacetylases, GSK-3beta, WNT/beta-catenin) to directly and indirectly influence many important biochemical pathways, low doses of lithium ions could have a significant impact on such processes. Support for this concept can be found in some reports of the associations between lithium-ion levels in water supplies and the incidence of dementias such as Alzheimer’s disease [43,221,222,223], or suicides [224,225]. Given the fact that some features of lithium ion effects exhibit sex differences [9,47], and some conditions such as dementia, cardiovascular disease, osteoporosis, and aspects of obesity/weight gain are prevalent in post-menopausal females [42,226], studies assessing the impact of low doses of lithium ions could be envisioned, using doses with very low potential for side effects. Such a direction is supported by reports that the lithium-responsive pathway involving GSK-3 is involved in Alzheimer’s disease [44,227,228]. Perhaps adding a low-dose lithium intervention with an effective exercise protocol would lead to enhanced outcomes, by potentially impacting complementary biological systems in a preventative manner to alleviate the development and progression of dementia [229].

The finding of coat color influences on the lithium-ion response pattern in mice, coupled with reports of racial differences in lithium-ion response patterns [137,138], could also point to new directions for the study of affective disorders in patients using some of the murine models reviewed earlier. For such investigations, one could develop the models in mice of differing coat colors and genetic backgrounds to assess the impact of background genes. In this regard, the role of melanocortins, specifically melanocortin-4 and its receptors, and the association with leptin pathways, could be explored in some detail. In this context, potential species differences (i.e., between mice and humans) could be a valuable tool rather than a stumbling block regarding the translation of information.

Relevant to the above concept is the fact that only 30% of bipolar patients treated with high doses of lithium can be considered “responders” [12,13,14]. Therefore, 70% of patients diagnosed with bipolar disorder according to current diagnostic criteria either have a different underlying disease mechanism operative compared to the 30% (discussed in [31]), or for unknown reasons, 70% of humans are resistant to lithium actions. Why 70% of humans would be resistant to doses that would likely have never been encountered from environmental exposure is certainly a conundrum that needs explanation. If 70% of *Homo sapiens* are resistant to aspects of lithium action, how this arose and why it is maintained in the population is not clear. Similarly, why some inbred strains of mice are also responsive to some aspects of lithium actions while others are not could provide some insights into the responder/non-responder conundrum in humans, building on studies such as those of Walss-Bass and Fries [190], Marie-Claire et al. [191,192], Zafrilla-Lopez et al. [196], and Pisanu et al. [195]. As inbred mouse strains are genetically restricted and humans are very heterogenous genetically, it would be potentially possible to narrow the search for genetic influences regarding lithium responsiveness/resistance, and then focus on translating the findings to different families exhibiting resistance or sensitivity to lithium exposure. Such studies may also provide insights into any organ-specific (i.e., renal vs brain; polydipsia vs bipolar disorder). This approach may also lead to insights into ethnic or racial differences in lithium sensitivity, and whether such phenotypes are associated with risk for disease, particularly those that are age-related.

While keeping the above concept in mind, perhaps further investigation of the well- established NZB/W model is also warranted. The mechanistic integration of the findings regarding the renal protection (i.e., GSK-3beta, WNT/beta-catenin, cyclic AMP), the polydipsia (i.e., cyclic AMP, GSK-3beta), coat color considerations (i.e., melanocortin/leptin pathway), obesity (i.e., leptin/melanocortin pathway), sex differences in lithium action (i.e., sex hormones and puberty-related events, GSK-3beta, leptin, and the immune dysfunction (i.e., leptin/melanocortin pathway, GSK-3beta, WNT/beta-catenin) attributes provide an attractive constellation of potential biomolecular mechanisms for assessing lithium actions in one model. Furthermore, extending the studies to involve areas of the brain and the heart could add some additional insights, as some patients with SLE develop central nervous system involvement, including psychiatric syndromes [230,231,232,233], and such changes are also reported in mouse models including the NZB/W model [102,234]. Patients with SLE are also at risk of cardiovascular disease [235,236], and such changes also can occur in the NZB/W model [103]. Thus, such an approach could lead to the evolution of new information about the inter-relationships regarding various organ involvement and how the molecular mechanisms may be overlapping.

In the proposed studies, the emphasis is partly on using lithium salts to dissect disease processes, but there would also be information generated which could be insightful, regarding how lithium serves as a relatively simple regulator of the biomolecular processes that arose through evolution, perhaps from single cell organisms to more complex organisms, such as humans.

## Figures and Tables

**Table 1:** Reported molecular targets of lithium action.

Molecules or Enzymes	
**Na/K ATPase**	**Adenyl Cyclase**
**Inositol Kinases and Phosphatases**	**GSK-3beta**
**Wnt/beta-catenin**	miRNAs
Histone Deacetylases	DNA Methylases
Cytokines and Growth Factors	Ion Channels
Bcl2 (upregulation)	Bax (downregulation)
Neurotransmitters	Cytoskeleton Components

A sample of the breadth of potential targets for lithium action. The bolded items represent molecules for which there are numerous references, including many of the reviews in Table 2 and Table 3, as well as those discussed in the text. The challenges for which effects are primary targets versus secondary targets relate to the dose and timing of lithium exposure, whether cells or in vivo studies were used, and the tissues assessed. While many of the above targets are considered primary targets, some may be secondary targets arising from impacting primary targets.
